# Effect of nebivolol on nitric oxide availability and ADMA concentration in CAD patients after PCI: Prospective randomized double-blind study

**DOI:** 10.5339/qmj.2026.55

**Published:** 2026-08-25

**Authors:** Ali A.R. Aldallal, Maryam A. Razzaq, Ahmed M. Abdul Hameed, Huda Jabber Merza, Hasanain Kamil Hasan, Salam Jasim Mohammed

**Affiliations:** 1 College of Pharmacy, Jabir Ibn Hayyan Medical University, Najaf, Iraq; 2 College of Pharmacy, Al-Mustaqbal University, Hillah, Iraq; 3 College of Medicine, University of Kufa, Kufa, Iraq

**Keywords:** Nebivolol, nitric oxide, asymmetric dimethylarginine, percutaneous coronary intervention, endothelial function

## Abstract

**Background:**

Endothelial dysfunction, defined by decreased bioavailability of nitric oxide (NO) and increased levels of asymmetric dimethylarginine (ADMA), has been proposed to play a central role in Coronary Artery Disease (CAD) pathophysiology. A selective β-blocker, nebivolol, could offer benefits in affecting this pathway after revascularization.

**Objective:**

The objective of this study is to determine the influence of nebivolol on serum NO levels and ADMA in patients with CAD who underwent successful elective percutaneous coronary intervention (PCI).

**Methods:**

In this prospective randomized double-blind placebo-controlled clinical study, 172 subjects with confirmed obstructive CAD and scheduled for PCI were recruited. After successful revascularization, patients were randomized to receive nebivolol 5 mg once daily (*n* = 89) or placebo (*n* = 83) for 42 days. Serum NO (μmol/L) and ADMA (ng/mL) were assessed at baseline and day 42 by colorimetry and ELISA, respectively. Paired t-tests and Analysis of Covariance (ANCOVA) analyses were performed for comparisons within and between the groups, respectively, after adjustment for baseline values.

**Results:**

The baseline clinical parameters and biomarker values were found to be equivalent between groups. After 42 days of therapy, there was a marked increase in serum NO levels among the nebivolol group (from 85.25 ± 9.8 to 128.47 ± 6.2; *p* < 0.001), whereas there was no change in the placebo group (*p* = 0.85). Simultaneously, there was a decrease in the levels of ADMA in the nebivolol-treated subjects (from 169.68 ±12.8 to 153.70 ±12.7; *p* < 0.001) and no change in the placebo-treated group. The ANCOVA analysis between the two groups for both markers was found to be highly significant at Day 42 (*p* < 0.001), independent of concomitant medications or baseline profiles.

**Conclusion:**

In patients after PCI, treatment for 42 days was found to significantly enhance NO availability and decrease systemic ADMA concentration by nebivolol. These results clearly establish that nebivolol has the potential to restore vascular homeostasis in the high-risk vascular window, hence making it an ideal supplementary treatment for patients having atherosclerotic diseases.

## 1. INTRODUCTION

Coronary artery disease is the most common type of cardiovascular disease observed worldwide and is still one of the leading causes of morbidity and mortality in developing and developed countries.^1^ CAD is basically caused by the progression of atherosclerosis, leading to the reduction in the coronary arteries and subsequent ischemia of the myocardial tissue.^2^ The prevalence of CAD has shown a significant increase of 30-60% in developed countries and is expected to be rising drastically in developing countries.^3^

Nitric oxide (NO) plays a crucial role in the functioning and integrity of the endothelial lining and has a preventive role against hypertension, atherosclerosis, and other forms of cardiovascular disease.^4^ NO is generated from the amino acid L-arginine by the enzyme endothelial nitric oxide synthase (e-NOS) present in the endothelial cells, catalyzing the conversion of arginine into NO and citrulline.^5^ NO levels can directly depend on the rate of its production but also on the influence of oxidative stress, wherein the superoxide free radicals can easily metabolize NO, especially in low doses or levels of L-arginine.^6^

Nebivolol is a long-acting, highly selective beta-1 receptor blocker classified as third generation and has antioxidant properties with the mechanism of NO-mediated vasodilation due to the beta-3 receptor stimulatory action.^7^ This beta-3 mechanism for nebivolol differentiates it from other beta-blockers, the vasodilatory beta-blockers carvedilol and labetalol, or the non-vasodilatory beta-1 blockers like the non-selective beta-blocker atenolol.^8^ Nebivolol cannot act on the membrane-stabilizing or sympathomimetic actions. However, clinical studies on nebivolol apply in the treatment of chronic heart failure and are used clinically in the US for hypertension, alone or in combination with other antihypertensive agents.^9^

Increased asymmetric dimethylarginine (ADMA), a natural inhibitor of e-NOS, has been related to cardiovascular risk.^10^ High levels of ADMA have been observed in many cardiometabolic diseases, such as diabetes mellitus,^11^ hypertension,^12^ peripheral vascular disease,^13^ stroke,^14^ pre-eclampsia,^15^ and atherosclerosis.^16^

Although there are proven advantages of nebivolol use theoretically, very few studies have been conducted on its role in mitigating the inhibitory effects of ADMA in the weeks post-percutaneous coronary intervention (PCI). The current study was a prospective randomized double-blind placebo-controlled trial to assess if nebivolol treatment at a dose of 5 mg/day for a period of 42 days increases the level of systemic NO and decreases ADMA concentrations in patients undergoing percutaneous coronary PCI.

## 2. METHODS

### 2.1. Study design and setting

This prospective, randomized, double-blind, placebo-controlled clinical trial was conducted at the Cardiothoracic Center, Al-Sadr Teaching Hospital, Al-Najaf, Iraq, from April to September 2024. The study protocol was approved by the Institutional Review Board of Jabir Ibn Hayyan Medical University (IRB No: JMU-PH/2024/23) and was conducted in accordance with the Declaration of Helsinki. Written informed consent was obtained from all participants.

### 2.2. Participants and eligibility

The age group considered varied between 34–70 years and comprised angiographically proven CAD and elective PCI.

#### Inclusion Criteria

- Verified obstructive CAD with at least one vessel ≥ 70% stenosis.
- Successful elective PCI with Drug-Eluting Stent (DES) placement.
- Baseline blood pressure stable above 110/70 mmHg.
- Patients who are currently not taking β-blockers with vasodilatory properties, such as nebivolol.

#### Exclusion Criteria

- History of primary pulmonary hypertension (which biases NO).
- Supplements like L-arginine that act on the NO pathway.
- Procedural complications documented in PCI (such as peri-procedural myocardial infarction, unstable angina, or decompensated heart failure).

It should also be acknowledged that PCI was successful in all the subjects and none of them had a procedure-related MI [Type 4a Myocardial Infarction (MI)] during the 42-day period.

### 2.3. Randomization and blinding

Participants were randomized using a simple random allocation method with an intended 1:1 ratio to receive either nebivolol (5 mg once daily) or a matching placebo for a duration of 42 days. Randomization was completed without block restriction. As a result of simple randomization, a minor imbalance in group sizes occurred by chance, with 89 participants allocated to the nebivolol group and 83 to the placebo group. Baseline characteristics were comparable between groups, demonstrating that the detected difference in group size was unlikely to have influenced the study results. The study participants and investigators remained blinded throughout the study. The patients received routine medical therapy with antiplatelet medications, statins, and Angiotensin-Converting Enzyme (ACE) inhibitors/ARBs, according to the existing guidelines of the European Society of Cardiology (ESC)/American Heart Association (AHA).

#### Nebivolol group

Has taken 5 mg of nebivolol orally once daily for 42 days.

#### Placebo group

Participants received the matching placebo once daily for 42 days.

All patients remained on routine medications for CAD, as usual, and as dictated by their attending cardiologists, with aspirin, clopidogrel, statins, ACE inhibitors or Angiotensin II Receptor Blockers (ARBs), and nitrates as needed.

### 2.4. Blood sampling and biomarker measurement

Blood samples were taken twice for each participant at 8:00 AM after an overnight fast. The patients were asked to follow a low-nitrate diet for 24–48 hours before blood collection:

- Baseline — prior to PCI and initiation of study medication.
- Day 42 — after completion of treatment.

### 2.5. Laboratory measurements

Venous blood (5 mL) samples were centrifuged at 3000 rpm for 15 minutes at 4°C. The separated serum was instantly aliquoted and stored at −80°C to certify the stability of the reactive metabolites.

***Serum nitric oxide (NO):*** NO concentrations were indirectly estimated by measuring the amount of nitrite present in the samples by performing a colorimetric Greiss reaction (Sigma-Aldrich). Greiss reactions consisted of a ‘reduction step‘, where nitrate was reduced to nitrite. The samples were deproteinized to eliminate any interactions and then analyzed by measuring the optical density at 540 nm with the help of a standard curve to determine concentrations in μmol/L.

***ADMA assays:*** Serum levels of ADMA were determined in terms of ng/mL by employing a highly sensitive commercial sandwich Enzyme-Linked Immunosorbent Assay (ELISA) kit (Elabscience, China) on a microplate reader after strict adherence to the manufacturer’s procedure, which involves absorption measurements taken at 450 nm wavelengths.

### 2.6. Research outcomes

#### Primary endpoints

- Serum levels of NO change between baseline and day 42.
- Serum levels of ADMA change between baseline and day 42.

#### Secondary endpoints

- Differences between groups regarding NO and ADMA levels on day 42.
- Associations of biomarker changes with demographics, comorbidities, and concomitant CAD therapies [Angiotensin-Converting Enzyme Inhibitors (ACEIs), ARBs, nitrates, statins].

### 2.7. Statistical analysis

The data analysis process was carried out using IBM Statistical Package for Social Sciences (SPSS) Statistics Version 25. Continuous data are represented with mean and standard deviation for greater biological variability. The results for the categorical data are shown in frequency and percentage. The Shapiro-Wilk test has been used to check the normality of data.

- ***Within-group analysis:*** The change from baseline to day 42 was evaluated using the paired-samples t-test.
- ***Between-groups comparison:*** To account for potential differences at baseline and the effect of co-existent standard medical treatment, such as ACE inhibitors and statins, the analysis conducted was Analysis of Covariance (ANCOVA). The day 42 measurement served as the response variable, and the independent variable was the group, with the baseline measurement used as the covariate.
- ***Categorical data:*** Differences in demographics and cardiovascular risk factors were evaluated using the Chi-square (χ^2^) test or Fisher’s exact test as appropriate.
- Significance: All tests were two-tailed, and a *p*-value < 0.05 was considered statistically significant.

## 3. RESULTS

### 3.1. Baseline characteristics

A total of 172 patients with proven CAD on coronary angiogram undergoing PCI were recruited and randomly allocated to treatment with nebivolol (*n* = 89) or placebo (*n* = 83). The average age of the participants was 57.45 ± 7.45 years, with a prevalence of male dominance of 79.1%. Cardiovascular risk factors identified in the group included 54.1% smokers, 67.4% hypertensives, and 61.6% with diabetes mellitus.

There was no statistically significant difference in the variables of age, gender, body mass index (BMI), smoking, hypertension, diabetes mellitus, associated ACEIs, ARBs, nitrates, statins, and number of blocked coronary arteries in the nebivolol and placebo groups (all p values > 0.05). These results indicate fruitful randomization and comparable baseline profiles between groups (Table 1).

### 3.2. Impact of nebivolol on NO

The values of NO at baseline were similar between nebivolol and placebo treatment (*p* = 0.85). After 42 days of treatment, nebivolol caused a highly significant increase in serum NO levels (*p* < 0.001), but there were no significant changes in serum NO levels in the placebo treatment. A highly significant difference was found between both treatment values at day 42 (*p* < 0.001), as shown in Table 2.

### 3.3. Role of nebivolol in reducing ADMA.

The baseline levels of ADMA were not significantly different among the groups (*p* = 0.47). On the 42nd day, there was a significant decrease in ADMA levels due to nebivolol (*p* < 0.001), whereas there was no significant change in the placebo group. The difference between the two groups at follow-up was significant (*p* < 0.001), as shown in Table 3.

### Summary of findings

NO levels were markedly increased, and ADMA was reduced following 42 days of nebivolol therapy.

- There were no significant changes in the biomarkers with the placebo.
- Equivalent baseline parameters support that results can be attributed to treatment with nebivolol.

## 4. DISCUSSION

In this randomized, double-blind, placebo-controlled clinical study, it was found that treatment for 42 days with nebivolol (5mg daily) significantly improved blood bioavailability of circulating NO as well as reduced ADMA levels in patients with CAD who had PCI. These hemodynamic and biochemical changes were unaffected by traditional risk factors such as smoking status, diabetes mellitus, hypertension, or severity of coronary obstructions, thus supporting once again its drug-specific role.

### 4.1. The role of L-Arginine/ADMA/NO axis after PCI recovery

Endothelial dysfunction remains physiologically the hallmark of CAD, which is distinguished by a basic imbalance between vasodilators, most notably NO, and vasoconstrictor factors.^4,17–19^ NO, which is catalyzed from L-arginine by e-NOS, is considered to be a master regulator of vasodilation, in addition to preventing platelet adhesion (5,6). During the high-risk period post-PCI, the implantation of stents in a biomechanical manner may trigger what is known as ‘endothelial stunning’, which is made worse by the competition of ADMA against e-NOS activity in its catalytic site. This leads to reduced activity of NO, creating a setting of oxidative stress.^10,16,20,21^

Our observation of the potent upsurge in NO levels post-nebivolol administration is consistent with the novel pharmacological action of nebivolol. Nebivolol is the only β-blocker that is a β_3_-adrenergic receptor agonist and directly activates e-NOS by the phosphoinositide 3-kinase (PI3K) signaling pathway.^9–11^ In addition, the substantial decrease in ADMA levels supports the theory that nebivolol increases the catabolism of ADMA, possibly through the modulation of Dimethylarginine Dimethylaminohydrolase (DDAH)expression and reduced oxidative degradation of the e-NOS cofactors.^20,21^

Clinically, the change in the biomarker reported herein argues for the additive effects of nebivolol beyond classical β-blocking activity. PCI by itself is accompanied by a temporary disruption of the endothelium, with consequent endothelial injury, inflammation, and reduced levels of NO bioavailability, at least in the short term.^2,3,17^ The ability of nebivolol to increase NO and reduce ADMA could thus independently stabilize the endothelium during the post-PCI period contributing to improving microvascular function, and more, potentially because our trial was not powered to evaluate major clinical outcomes such as restenosis, or major cardiovascular events, or death, with a negative consequence on cardiovascular health, particularly because the change in NO, and by extension, in ADMA, provides a plausibly mechanistic argument for improved cardiovascular health in such a high-risk population.^17,18,21,22^

Nevertheless, the results of this study contribute to the existing body of knowledge indicating that nebivolol is not just an effective β_1_-selective antagonist but also has beneficial effects on endothelial functions that might be useful in patients with coronary atherosclerosis.^7,9,23,24^ Longitudinal studies involving larger numbers of patients would be required to verify whether the observed augmentation in NO and reduction in ADMA lead to any clinical benefit.

### 4.2. Limitations

Though this randomized controlled trial yields strong scientific data regarding the influence that nebivolol exerts on NO and ADMA levels, some limitations should not be overlooked:

- Trial registration: Due to institutional constraints at the start of the study, the trial was not prospectively registered in a public clinical trials database. However, the study was conducted in strict adherence to a pre-defined protocol approved by the Local Ethics Committee and tracked the ethical principles of the Declaration of Helsinki to ensure data integrity.
- NO levels: NO levels were determined indirectly by the colorimetric Griess reaction. Although such a common assay has far-reaching uses, it mainly detects the presence of nitrites. Future studies involving chemiluminescence or High-Performance Liquid Chromatography (HPLC) analysis of total NOx concentrations would certainly yield a more comprehensive pattern regarding NO bioavailability.
- PCI context: Although every patient had successful PCI, the procedure provokes a transient inflammatory reaction. Although randomization between groups for nebivolol and placebo was attempted to neutralize this effect, the acute and chronic variations of endothelial biomarkers in response to stenting might affect the absolute concentration of the biomarker.
- Only patients with CAD and PCI were included, and there was no control group of non-CAD patients; thus, results cannot be generalized for non-CAD patients.
- Nebivolol was evaluated in patients not on vasodilatory β-blockers, but other medications (statins, ACE inhibitors/ARB inhibitors) could be continued, despite not showing significant imbalance in both groups, due to the possible minor effects on the endothelium.
- The study examined biochemical indexes for endothelial function and did not examine endpoints for long-term clinical success like the occurrence of restenosis, Major Adverse Cardiovascular Events (MACE), or assessment for endothelial function with imaging and flow-mediated dilation. Lastly, the study reflects results from a single center, which may limit broader applicability.

## 5. CONCLUSION

The treatment regimen of nebivolol (5 mg/day) has been noted to increase the bioavailability of NO and decrease the concentrations of ADMA in CAD patients following a PCI procedure. These outcomes established the promise of nebivolol in offering vascular protection rather than its use in managing blood pressure issues in patients. The drug targets the basic biochemical mechanisms of endothelial dysfunction; therefore, nebivolol is a superior drug in enhancing vascular remodeling after a coronary revascularization procedure.

## ACKNOWLEDGEMENT

The authors would like to express their sincere appreciation to the healthcare professionals and laboratory staff who assisted with patient recruitment, sample collection, and laboratory analyses. The authors also thank all participants for their valuable contribution to this study.

## AUTHORS’ CONTRIBUTIONS

All authors contributed substantially to the conception and design of the study, patient recruitment, data acquisition, data analysis, interpretation of the results, and manuscript preparation. All authors critically revised the manuscript, approved the final version, and agree to be accountable for all aspects of the work.

## CONFLICT OF INTEREST

The authors declare that they have no competing interests or conflicts of interest related to this work.

## DISCLOSURE OF AI USE

During the manuscript revision process, the authors used an AI-assisted language tool solely to improve the clarity, grammar, and readability of the text. The study design, data collection, data analysis, interpretation of the results, scientific conclusions, and all editorial decisions were performed exclusively by the authors, who take full responsibility for the content of the manuscript.

## DATA AVAILABILITY

The datasets generated and/or analyzed during the current study are available from the corresponding author upon reasonable request.

## FUNDING

This research received no external funding.

## ETHICAL APPROVAL

The study protocol was approved by the Institutional Review Board of Jabir Ibn Hayyan Medical University (IRB No: JMU-PH/2024/23).

## Figures and Tables

**Table 1.** Baseline characteristics of the study population.

Variable	Nebivolol (*n* = 89)	Placebo (*n* = 83)	*p*-value
Age (years)	56.71 ± 7.83	58.24 ± 7.01	0.488
Male (%)	71 (52.5%)	65 (47.8%)	0.814
Female (%)	18 (50.0%)	18 (50.0%)	0.814
BMI (kg/m^2^)	28.79 ± 4.53	27.95 ± 4.70	0.906
Smoking (%)	48 (51.6%)	45 (48.4%)	0.970
Hypertension (%)	57 (49.1%)	59 (50.9%)	0.325
Diabetes mellitus (%)	51 (48.1%)	55 (51.9%)	0.277
ACEIs use (%)	23 (57.5%)	17 (42.5%)	0.406
ARBs use (%)	27 (60.0%)	18 (40.0%)	0.197
Nitrate use (%)	51 (54.3%)	43 (45.7%)	0.469
Statin use (%)	27 (48.2%)	29 (51.8%)	0.520
Obstructed vessels			0.277
One vessel	39 (58.2%)	28 (41.8%)	
Two vessels	24 (43.6%)	31 (56.4%)	
Three vessels	26 (52.0%)	24 (48.0%)	

Values expressed as means ± SD or numbers with frequency; *p* < 0.05.

**Table 2.** Serum nitric oxide (NO) concentrations pre- & post-treatment.

Group	*N*	Baseline NO (µmol/L)	Day 42 NO (µmol/L)	*p*-value
Nebivolol	89	85.25 ± 9.8	128.47 ± 6.2	< 0.001*
Placebo	83	82.55 ± 10.5	84.28 ± 10.48	0.850

*Paired *t*-test for comparison within group and independent *t*-test revealed significance for difference among groups at day 42 (*p* < 0.001).

**Table 3.** Serum levels of ADMA before and after the treatment.

Group	*n*	Baseline ADMA (ng/mL)	Day 42 ADMA (ng/mL)	*p*-value
Nebivolol	89	169.68 ± 12.8	153.70 ± 12.7	< 0.001*
Placebo	83	165.07 ± 12.2	165.55 ± 11.84	0.466

*Paired *t*-test for within-group comparison. The results of the independent t-test revealed a significant difference on day 42 (*p* < 0.001).
